# Breast Cancer Metastasis to the Upper Arm: A Rare Case and Review of the Literature

**DOI:** 10.70352/scrj.cr.25-0036

**Published:** 2025-07-01

**Authors:** Yuka Nishimura, Hirofumi Terakawa, Shinji Miwa, Hiroko Kawashima, Hiroko Ikeda, Hiroshi Yoshino, Noriyuki Inaki

**Affiliations:** 1 Department of Breast Surgery, Kanazawa University Hospital, Kanazawa, Ishikawa, Japan; 2 Breast Center, Kanazawa University Hospital, Kanazawa, Ishikawa, Japan; 3 Breast and Endocrinological Surgery, Ishikawa Prefectural Central Hospital, Kanazawa, Ishikawa, Japan; 4 Department of Orthopaedic Surgery Graduate School of Medical Sciences, Kanazawa University, Kanazawa, Ishikawa, Japan; 5 Department of Diagnostic Pathology, Kanazawa University Hospital, Kanazawa, Ishikawa, Japan; 6 Department of Gastrointestinal Surgery/Breast Surgery, Kanazawa University Hospital, Kanazawa, Ishikawa, Japan

**Keywords:** soft tissue metastasis, lymph node metastasis, upper arm, breast cancer

## Abstract

**INTRODUCTION:**

Soft tissue metastasis of breast cancer is very rare. Specifically, metastasis to the upper arm has only been reported in 10 cases, including this one, to the best of our knowledge. Distant lymph node metastasis of breast cancer is also infrequent, with only one documented case of brachial lymph node metastasis.

**CASE PRESENTATION:**

We report a case of soft tissue or lymph node metastasis in the right upper arm, diagnosed 3 years after surgery for right breast cancer. The patient, a 79-year-old woman, was receiving postoperative hormone therapy for right breast cancer. She presented with edema and numbness in the right upper arm during a routine follow-up examination 3 years post-surgery. Ultrasonography revealed a 20 mm mass in the right brachial muscle, which was diagnosed as adenocarcinoma via fine-needle aspiration, suggesting breast cancer metastasis. Further examination showed that the mass was in close proximity to the nerves and veins, but no definitive evidence of invasion was observed. Surgical resection was performed. The tumor was particularly adherent to the nerve, complicating resection. While no gross residual tumor was noted, histopathological analysis indicated positive surgical margins. After surgery for soft tissue or lymph node metastasis, the patient continued hormone therapy postoperatively. However, approximately 1 year and 3 months later, tumor recurrence at the resection site and skin metastasis were observed. Consequently, hormone therapy was modified, and oral cyclin-dependent kinase 4/6 (CDK4/6) inhibitors were initiated.

**CONCLUSIONS:**

Metastasis of breast cancer to soft tissue and the brachial lymph nodes is extremely rare. Diagnosis may be delayed in asymptomatic cases, and when metastasis is in close proximity to surrounding structures, such as the nerves, histological diagnosis can be challenging. Metastatic soft-tissue tumors are associated with a poor prognosis; therefore, early diagnosis and appropriate multimodal treatment are crucial.

## Abbreviations

CDK4/6
: cyclin-dependent kinase 4/6
DISH
: dual *in situ* hybridization
ER
: estrogen receptor
FDG
: fluorodeoxyglucose
FNA
: fine-needle aspiration
HER2
: human epidermal growth factor receptor 2
IHC
: immunohistochemistry
MRI
: magnetic resonance imaging
PET-CT
: positron emission tomography-computed tomography
PgR
: progesterone receptor
RCB
: residual cancer burden
SUV
: standardized uptake value

## INTRODUCTION

Metastasis of breast cancer to soft tissues is extremely rare, with only 10 cases of upper arm metastasis reported to date, including the present case.^1–7)^ Brachial lymph node metastasis of breast cancer is also extremely rare, with only one documented case reported to the best of our knowledge.^8)^ Asymptomatic cases may result in delayed diagnosis. Additionally, diagnosing metastatic soft-tissue tumors or distant lymph node metastases is often challenging, particularly in cases like the present one, where the metastases are in close proximity to surrounding structures, such as the nerves, necessitating surgical biopsy for confirmation.^9)^ Metastatic soft-tissue tumors are associated with a poor prognosis.^3,4)^ Regardless of the case, early diagnosis and treatment are crucial, and appropriate multidisciplinary management is required. In this case, a recurrence of right breast cancer was observed in the upper arm 3 years after surgery, and the patient was treated for suspected soft tissue or lymph node metastasis. We present this case along with a literature review.

## CASE PRESENTATION

A 79-year-old woman had undergone a left mastectomy and axillary lymph node dissection for left breast cancer 37 years ago, though details of the procedure were unknown. Her sister had also developed breast cancer, but no further details were available. The patient was receiving medical treatment for hypertension, dyslipidemia, and dizziness. She had no history of alcohol consumption or smoking.

Three years ago, she was diagnosed with breast cancer (cT3N2aM0, cStage IIIA) in the right upper breast near the nipple. Immunohistochemical analysis revealed estrogen receptor (ER)-positive status (ER+, >95%, score 3b), progesterone (PgR)-positive status (PgR+, 30%, score 3a), human epidermal growth factor receptor 2 (HER2)-negative by immunohistochemistry (HER2-IHC, score 0), HER2-negative by dual *in situ* hybridization (HER2-DISH), and a Ki-67 index of 30%. A positron emission tomography-computed tomography (PET-CT) scan performed prior to treatment initiation showed no abnormal uptake suggestive of metastasis, including in the upper arm. The patient was informed about BRCA1/2 genetic testing but opted not to proceed after an informed discussion, and her decision was respected. Neoadjuvant chemotherapy was administered, consisting of 4 courses of epirubicin (90 mg/m^2^) and cyclophosphamide (600 mg/m^2^), followed by 4 courses of docetaxel (75 mg/m^2^). The tumor size reduced from 60 to 28 mm following treatment. She subsequently underwent a right mastectomy and Level II lymph node dissection. Pathological analysis revealed ypT2N3a, ypStage IIIC disease with ER-positive status (>95%, score 3b), PgR-negative status (<1%, score 1), HER2-negative (score 1+), HER2-DISH-negative, a Ki-67 index of 2.7%, and negative surgical margins. The residual cancer burden (RCB) was classified as RCB-II,^10)^ with metastases detected in 10 out of 16 lymph nodes. Postoperatively, the patient received adjuvant radiation therapy to the chest wall and supraclavicular region (2 Gy × 25 fractions = total 50 Gy). A 10-year course of hormone therapy was planned, and the patient was initiated on letrozole (2.5 mg/day), an aromatase inhibitor.

**Fig. 1:**
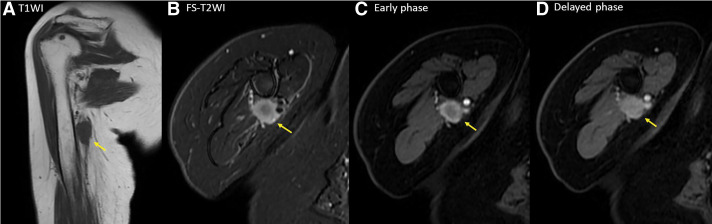
(**A**, **B**) MRI showing a well-defined mass with low signal intensity on T1-weighted imaging (coronal) and high signal intensity on fat-suppressed T2-weighted imaging (axial). (**C**, **D**) Contrast-enhanced MRI scan demonstrating strong contrast enhancement with rim predominance. T1WI, T1-weighted imaging; T2WI, T2-weighted imaging

**Fig. 2:**
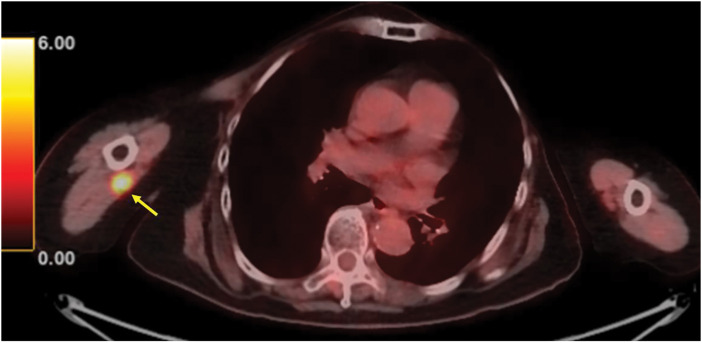
PET-CT axial and coronal images showing an FDG-avid lesion. PET-CT, positron emission tomography-computed tomography; FDG, fluorodeoxyglucose

The patient presented to our department for a follow-up examination 3 years after surgery. She reported experiencing edema and mild numbness in the right upper extremity for the past 6 months. No obvious mass was detected in the axilla; however, a 20 mm mass was palpable from the right axilla to the transition area of the upper arm. Ultrasonography confirmed the presence of a 20 mm mass in the same region. Magnetic resonance imaging (MRI) revealed a relatively well-defined 20 mm mass at the mid-level of the right upper arm, exhibiting low signal intensity on T1-weighted images and high signal intensity on fat-suppressed T2-weighted images. Contrast-enhanced MRI demonstrated a strong enhancement effect with a predominant rim enhancement pattern (**Fig. 1**). The right brachial vein and nerve were in close proximity to the mass. Ultrasonography and MRI showed no clear evidence of invasion into the surrounding tissues. However, the proximity of the median nerve, ulnar nerve, and brachial vein necessitated careful fine-needle aspiration (FNA). FNA of the mass confirmed adenocarcinoma, suggesting metastatic breast cancer. PET-CT demonstrated fluorodeoxyglucose (FDG) accumulation within the mass, with a maximum standardized uptake value (SUVmax) of 6.1 in the early phase and 7.3 in the delayed phase. No other distant metastases were detected (**Fig. 2**). Tumor markers were within the normal range.

**Fig. 3:**
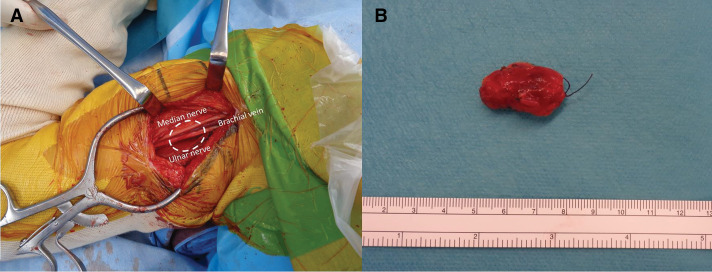
(**A**) Intraoperative findings showing no gross residual tumor. (**B**) Resected specimen.

**Fig. 4:**
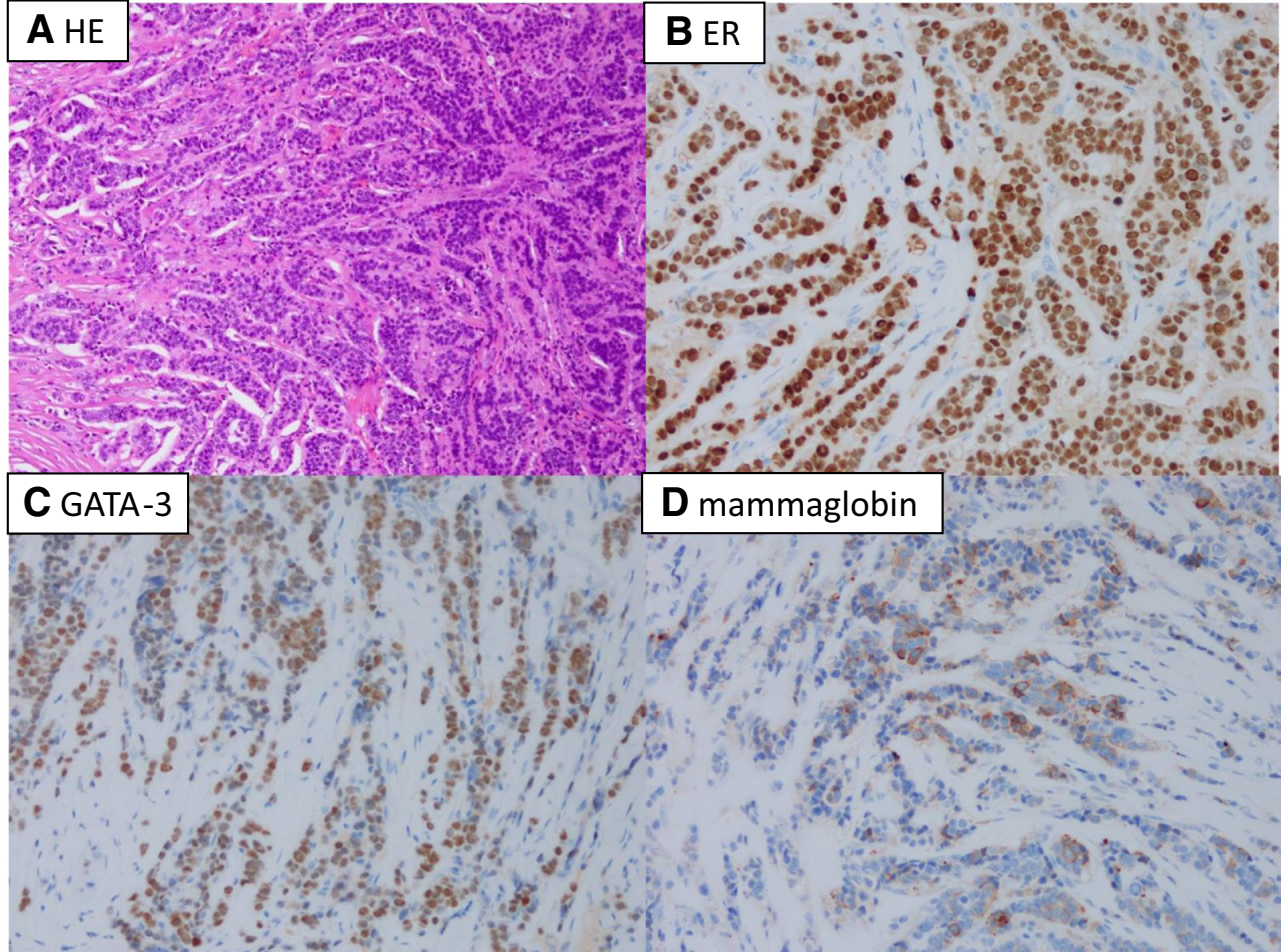
Pathological findings of the resected specimen. (**A**) Hematoxylin and eosin staining (×40). (**B**–**D**) Immunostaining showing ER and GATA-3 positivity, with weak mammaglobin positivity (×40).

Based on ultrasonography and MRI findings, no invasion into the surrounding tissues was detected, and surgical treatment was planned. The patient underwent tumor resection in the orthopedic surgery department. The operation lasted 1 h and 40 min, with minimal intraoperative bleeding. As indicated by preoperative imaging, the tumor was located in the anterior border of the medial head of the triceps brachii muscle, with invasion into the anterior adipose tissue. The tumor was in close proximity to the median nerve, ulnar nerve, and brachial vein and was particularly adherent to the nerves, making resection challenging. The tumor was located in the anterior border of the medial head of the triceps brachii muscle, with invasion into the anterior adipose tissue. No suspicious lymphatic structures were identified during surgery. Gross examination revealed no residual tumor (**Fig. 3**). Histopathological analysis confirmed adenocarcinoma with histological features similar to those of the primary breast cancer. Immunohistochemical staining demonstrated ER-positive status, GATA-3 positivity, and weak mammaglobin expression (**Fig. 4**). Although GATA3 is known as a marker for breast cancer, its sensitivity and specificity are not 100%. In addition to breast cancer, GATA3 positivity has been reported in other tumors such as urothelial carcinoma, renal cell carcinoma, gynecological tumors, salivary gland tumors, hematologic malignancies, and skin cancers. However, in the present case, since PET-CT ruled out tumors in other organs, the lesion was diagnosed as a metastasis of breast cancer. Perineural invasion and vascular invasion were observed within the tumor, with infiltration into the surrounding adipose and muscle tissues. The surgical margins were positive. No lymph node involvement was identified. The patient was diagnosed with postoperative recurrence of right breast cancer, presenting as either soft tissue metastasis or lymph node metastasis in the upper arm.

**Fig. 5:**
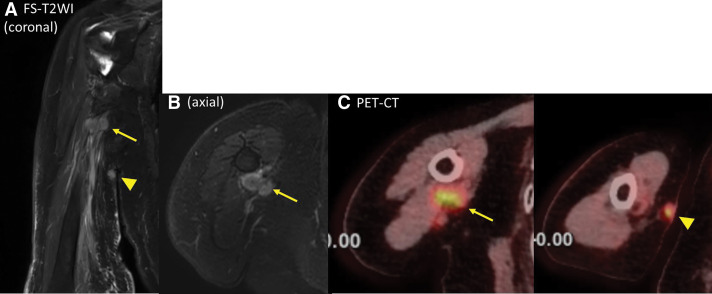
(**A**, **B**) MRI (fat-suppressed T2-weighted imaging) scans taken 1 year and 3 months after the initial surgery. (**C**) PET-CT scan taken 1 year and 3 months after the initial surgery. A mass was observed at the tumor resection site (arrow), and skin metastasis was also identified (triangle). T2WI, T2-weighted imaging; PET-CT, positron emission tomography-computed tomography

Postoperatively, mild numbness persisted in the right upper extremity, but edema improved. The addition of radiotherapy and an escalation to CDK4/6 inhibitors or chemotherapy were considered; however, based on the patient’s activities of daily living and personal preferences at that time, neither radiotherapy, CDK4/6 inhibitors nor chemotherapy were administered as postoperative therapy. Postoperative letrozole therapy was continued. Approximately 1 year and 3 months after surgery, recurrence at the tumor resection site on the upper arm and skin metastasis was observed (**Fig. 5**). Tumor markers remained within the normal range, and no new symptoms developed. Hormonal therapy was switched to fulvestrant (500 mg/month), and abemaciclib (300 mg/day), a CDK4/6 inhibitor, was initiated. Radiation therapy will be considered in the future based on symptom progression.

## DISCUSSION

This case report describes a postoperative recurrence of breast cancer in the ipsilateral upper arm, presenting as either soft tissue or lymph node metastasis.

Metastasis of primary breast cancer to soft tissues is rare.^5)^ To the best of our knowledge, only 10 cases of upper arm metastasis from breast cancer have been reported, including the present case^1–7)^ (**Table 1**). The incidence of soft tissue metastases has been reported as 0.8% in autopsy cases^11)^ and 0.2% in clinical cases.^12)^ This rarity is believed to result from the production of anti-carcinogenic factors by soft tissues, including lactic acid, β-adrenergic receptors, and protease inhibitors, which act as inhibitory agents against metastasis and invasion.^13–15)^ Additionally, changes in pH, the accumulation of metabolites, and local temperature variations at soft tissue sites have been reported as contributing factors.^14)^

**Table 1:** Case series and case report of breast cancer metastatic to soft tissue of arm

Author/Year	No of cases	Age at the time of metastasis	Site	Time between breast cancer and metastasis	Treatment	Survival time
Plaza/2008	3/118^*^	67	Arm	NA	NA	NA
		48	Arm	NA	NA	NA
		77	Forearm	NA	NA	NA
Konatam/2015	1	26	Forearm	Simultaneously	Chemotherapy with doxorubicin and cyclophosphomide	NA
Ogiya/2015	1/13^**^	Unknown	Forearm and Bone	NA	NA	NA
Liu/2015	1	65	Forearm, Psoas, Quadratus Lumborum	7 months	Surgical excision for symptomatic relief	Died 12 months postoperatively
Purkayastha and Shrma/2016	1	65	Arm and Shoulder	15 years	Local palliative radiotherapy	Died
Almusarhed and Eldeeb/2017	1	60	Forearm	25 years	Letrozole and palliative radiotherapy (15 fr/40.05 Gy)	>6 months
Omranipour/2021	1	31	Forearm	6 years	Local excision, 6 courses of cyclophosphamide and docetaxel, tamoxifen	>3 years
Nishimura/2022	1	79	Arm	3 years	Local excision, anastrozole	>6 months

^*^Total number is soft tissue metastasis reported with different origin.

**Total number is muscle metastasis from breast cancer.

NA, not applicable

Plaza et al.^1)^ analyzed 118 cases of soft tissue metastasis and found that breast cancer accounted for 13 cases/11%, skin cancer for 16%, lung cancer for 11%, colon cancer for 10%, and kidney cancer for 10%.

While bone, lung, liver, brain, and lymph nodes are the most common sites of breast cancer metastasis, soft tissue metastases remain exceedingly rare.^5)^ Plaza et al.^1)^ and Torigoe et al.^16)^ reported that the anterior abdominal wall was a frequent site of soft tissue metastasis, while Damron et al.^17)^ identified the thigh and Surov et al.^14)^ described metastases in the extraocular muscle. Other common metastatic locations include the back, chest wall, and lower limbs, with the upper limb being the least frequently affected site, as observed in this case.

The differential diagnosis of soft tissue lesions includes soft tissue sarcoma and lymphangiosarcoma, both of which can cause lymphedema.^5,17)^ Lymphedema is a well-recognized complication of breast cancer treatment, occurring after breast cancer surgery or radiotherapy, and can present with similar clinical manifestations. Reported cases of upper arm metastasis from breast cancer have shown long intervals between the initial breast cancer diagnosis and the identification of metastases, with durations extending up to 25 years.^6)^ Some cases have been identified during physical therapy sessions, highlighting the importance of careful and thorough clinical examinations in facilitating early detection.^7)^

MRI is the primary imaging modality for diagnosing soft tissue metastases. Metastatic lesions exhibit higher FDG uptake than normal tissue, making PET-CT scans a sensitive and valuable tool for detection.^18)^ A histopathological examination is essential for a definitive diagnosis. In this case, the diagnosis was confirmed via FNA. If the tumor is located in the superficial layer and the surrounding neural structures are clearly delineated, a percutaneous ultrasound-guided approach is feasible. However, depending on the tumor size and its anatomical relationship with adjacent structures, a surgical biopsy may be preferable.^9)^

Upper arm lymph node metastasis is also rare and, to the best of our knowledge, has only been reported in 1 case.^8)^ In that case, the metastasis was diagnosed as brachial lymph node involvement because lymph node structures were observed in the vicinity of the tumor cells. Several mechanisms have been proposed to explain metastasis to the brachial lymph nodes. Lymphatic flow from the breast is primarily directed toward the axillary, lateral mammary gland, sternoclavicular joint, and internal mammary pathways.^19)^ One hypothesis suggests that at the time of the initial breast cancer surgery, the axillary, supraclavicular, subclavian, and internal mammary lymph nodes were already infiltrated with tumor cells, predisposing the brachial lymph nodes to later metastasis. Another possibility is that postoperative radiation therapy altered lymphatic drainage, leading to redirection of the lymphatic flow to the upper arm and subsequent metastasis to the brachial lymph nodes. Additionally, pre-existing micrometastases in the brachial lymph nodes at the time of surgery may have progressed due to the absence of radiation therapy targeting the upper arm. According to the latest (eighth) edition of the American Joint Committee on Cancer classification, regional lymph nodes include ipsilateral axillary lymph nodes (Levels I–III), ipsilateral supraclavicular lymph nodes, and ipsilateral internal mammary lymph nodes.^20)^ By contrast, metastases to non-regional lymph nodes, such as contralateral intramammary or contralateral axillary lymph nodes, are classified as distant metastases, similar to soft tissue metastases.^21)^

Regarding the treatment of metastatic tumors, multimodal therapy is typically tailored based on the patient’s performance status, comorbidities, primary tumor type, and the location and size of metastatic lesions.^22)^ Torigoe et al.^16)^ and Damron et al.^17)^ reported 1-year survival rates of 47% and 25%, respectively, indicating that the prognosis for patients with soft tissue metastases remains poor.

Oligometastatic disease is defined as the presence of a limited number of metastatic lesions on systemic imaging. However, no universally accepted definition exists. The generally accepted criterion for oligometastasis is 5 or fewer metastases confined to a single organ, though some studies define it as fewer than 5 metastases with a maximum lesion size of less than 5 cm.^23,24)^ Oligometastatic disease is considered as an intermediate state between localized disease and widespread systemic dissemination. In this case, the patient developed a recurrence. However, if the primary tumor is well controlled or surgically resected, and metastatic lesions are also removed, the disease-free period may be prolonged, potentially leading to complete remission.^25)^ The efficacy of radiation therapy in the management of oligometastases has also been well documented. Specifically, patients under 55 years of age, those with hormone receptor-positive tumors, limited bone or liver metastases, low-grade tumors, good performance status, a long disease-free interval (>12 months), and a favorable response to systemic therapy are most likely to benefit from local interventions such as surgery or radiation therapy.^26)^

In this case, the tumor was surgically resected; however, the surgical margins were positive. The tumor recurred within a short period, and the patient is currently receiving systemic drug therapy for metastatic breast cancer. Radiation therapy is planned based on symptom progression, including pain management.

Soft tissue metastasis and brachial lymph node metastasis of breast cancer are extremely rare, and diagnosis may be delayed, particularly in asymptomatic patients. It is crucial to consider these entities in the differential diagnosis, perform a thorough clinical evaluation, and select appropriate diagnostic tests to confirm the diagnosis. Multidisciplinary treatment should be tailored to the individual patient’s clinical condition. If oligometastatic breast cancer is treated, close monitoring is essential to detect recurrence early.

## CONCLUSIONS

Soft tissue metastasis and lymph node metastasis of breast cancer in the upper arm are extremely rare. Diagnosis may be delayed in asymptomatic cases, and when metastases are in close proximity to surrounding structures, such as the nerves, histological diagnosis can be challenging. Early diagnosis and timely intervention are crucial for managing both metastatic soft-tissue tumors and distant lymph node metastases. Individualized, multidisciplinary treatment selection is essential for optimizing patient outcomes.

## ACKNOWLEDGMENTS

The authors would like to thank Nature Research Editing Service (http://bit.ly/NRES_BS) for English language editing.

## DECLARATIONS

### Funding

Not applicable.

### Authors’ contributions

YN is the first author and drafted the manuscript under the supervision of HT, SM, HK, HI, NI, and HY.

All authors reviewed and approved the final version of the manuscript.

### Availability of data and materials

Not applicable.

### Ethics approval and consent to participate

This work does not require ethical considerations or approval. Informed consent to participate in this study was obtained from the patient.

### Consent for publication

Written informed consent was obtained from the patient for the publication of this case report and any accompanying images.

### Competing interests

The authors declare that they have no competing interests.
